# Quality of life impairment associated with body dissatisfaction in a general population sample of women

**DOI:** 10.1186/1471-2458-13-920

**Published:** 2013-10-03

**Authors:** Jonathan Mond, Deborah Mitchison, Janet Latner, Phillipa Hay, Cathy Owen, Bryan Rodgers

**Affiliations:** 1 Research School of Psychology, Australian National University, Canberra ACT 0200, Australia; 2 School of Medicine, University of Western Sydney, Campbelltown, Australia; 3 Department of Psychology, University of Hawaii at Manoa, Honolulu, USA; 4 School of Medicine, University of Western Sydney, Campbelltown, Australia; 5 School of Medicine, James Cook University, Townsville, Australia; 6 Rural Clinical School, Medical School, Australian National University, Canberra, Australia; 7 Australian Demographic & Social Research Institute, Australian National University, Canberra, Australia

**Keywords:** Body dissatisfaction, Prevalence, Disability, Quality of life impairment, Health burden

## Abstract

**Background:**

In order to elucidate the individual and community health burden of body dissatisfaction (BD), we examined impairment in quality of life associated with BD in a large, general population sample of women.

**Methods:**

Self-report measures of BD, health-related quality of life (SF-12 Physical and Mental Component Summary scales) and subjective quality of life (WHOQOL-BREF Psychological Functioning and Social Relationships subscales) were completed by 5,255 Australian women aged 18 to 42 years.

**Results:**

Most participants (86.9%) reported some level of dissatisfaction with their weight or shape and more than one third (39.4%) reported moderate to marked dissatisfaction. Higher levels of BD were associated with poorer quality of life for all items of both quality of life measures, the degree of impairment being proportional to the degree of BD. Associations were strongest for items tapping mental health and psychosocial functioning, although greater BD was associated with substantially increased risk of impairment in certain aspects of physical health even when controlling for body weight. Post-hoc analysis indicated that the observed associations between BD and quality of life impairment were not accounted for by an association between BD and eating disorder symptoms.

**Conclusions:**

In women, BD is associated with marked impairment in aspects of quality of life relating to mental health and psycho-social functioning and at least some aspects of physical health, independent of its association with body weight and eating disorder symptoms. Greater attention may need to be given to BD as a public health problem. The fact that BD is “normative” should not be taken to infer that it is benign.

## Background

In epidemiologic and public health research, non-fatal health conditions are deemed to constitute a public health problem to the extent that they are both prevalent and disabling [1]. Disability is often assessed using one or more measures of health-related quality of life, that is, measures of the perceived effect of an individual’s health on his or her everyday functioning [2,3], although individuals’ subjective satisfaction with different facets of their lives - including, but not limited to, their health status - is considered equally important by many authorities [4,5]. Individuals’ subjective satisfaction with their lives may be referred to as subjective quality of life, although it needs to be recognized that all quality of life measures are subjective to some extent and that there are no universally accepted definitions of such terms [5,6]. Both health-related quality of life and subjective quality of life have been found to be strongly predictive of more objective indices of health status, including chronic disease and mortality, as well as health service utilization, hence their utility as measures of disease burden [7-9].

There is no doubt that body dissatisfaction (BD) is prevalent. Findings from epidemiological studies have consistently shown that many, if not most, younger women in industrialized nations are at least moderately dissatisfied with their body weight or shape [10]. The term “normative discontent” was introduced in the 1980’s to describe the pervasiveness of this phenomenon and it is no less apposite today [10,11]. BD remains less common in men, although the gap may be closing [10,12].

Whether and to what extent BD should be considered disabling is less clear. On the one hand, there is good evidence that BD is associated with - and predictive of - a range of adverse health outcomes, including low self-esteem, depressive mood and eating disorder symptoms [13-15]. There is also good evidence that BD mediates the association between obesity and emotional well-being, in young women at least [12,16]. On the other hand, attention has focused, almost without exception, on the status of BD as a risk factor for other, “more serious” mental health problems rather than as a public health problem in its own right [13,17,18]. As a consequence, little is known about the effects of BD on quality of life. Given the demonstrated links between BD and impairment in emotional well-being [13-15], adverse effects of BD on quality of life might be expected to be most pronounced for items tapping perceived impairment in mental health and psycho-social functioning, whereas impairment in physical health might be expected to be less pronounced and due, at least in part, to the positive association between BD and body weight [12]. However, the available evidence does not permit any firm conclusions in this regard.

Meland and colleagues [19] found, in a large, general population sample of adolescents, that perceived negative health was more common among girls than among boys and that this was accounted for by higher levels of BD - and a stronger association between BD and perceptions of health - among girls. However, findings from this study are difficult to interpret because the assessment of perceived health was confined to a single item, namely, “How healthy do you think you are?”, that presumably encompassed perceived impairment in both physical and mental health [20]. Further, no attempt was made to control for body weight in exploring the associations between BD and perceptions of health.

Muennig and colleagues [21] found, in a general population sample of women and men, that the difference between actual and desired weight was a better predictor of self-reported “unhealthy” days in the past month than actual body weight and that this was the case for both physical and mental health. Further, and consistent with the findings of Meland et al. [19], poor self-reported health was more common in women than in men and this was accounted for, at least in part, by higher levels of BD, and a stronger association between BD and perceived impairment, in women.

Of note is that there was no assessment of eating disorder symptoms, namely, the “undue influence of weight or shape on self-evaluation” and the occurrence of binge eating and/or extreme weight-control behaviors [20,22], in either of these studies. Given the strong links between BD and eating disorder symptoms [18], and given that eating disorder symptoms are associated with marked impairment in quality of life [20,22-24], it would be helpful to consider the potential role of these symptoms in accounting for any observed associations between BD and quality of life impairment. In a community-based study of quality of life impairment associated with eating disorder symptoms in women, Vallance and colleagues [25] found moderate negative correlations between BD and both physical and mental component summary scales of the (36-item) Medical Outcomes Study Short Form (SF-36) in preliminary (bivariate) analysis. Whether these associations might have been due to an association between BD and eating disorder symptoms was not, however, considered.

To our knowledge, no other research has examined the association between BD and impairment in quality of life in a general population sample. This is regrettable because population-based research addressing impairment in quality of life associated with BD, when taken with findings relating to prevalence, has the potential to highlight the significance of BD as a public health problem - and, in turn, the need for a public health response - in the same way that findings from epidemiological studies of disability associated with the more common mental health problems have highlighted the public health burden of these conditions [3,23,26].

The goal of the present study was, therefore, to examine impairment in quality of life associated with the spectrum of BD that occurs at the population level, using measures of both health-related and subjective quality of life. Although the existing evidence did not permit any firm *a priori* hypotheses, it was reasonable to surmise that adverse effects of BD on quality of life would be most pronounced for items tapping perceived impairment in or dissatisfaction with mental health and psycho-social functioning, whereas impairment in physical health associated with BD would be expected to be less pronounced and due, at least in part, to the association between BD and body weight. A secondary aim of the study was to determine whether any observed associations between BD and quality of life impairment could be accounted for by an association between BD and eating disorder symptoms.

## Methods

### Study design and participants

The research was conducted as part of the *Health and Well*-*Being of Female ACT Residents Study*, an epidemiological study of disability associated with eating disorder symptoms among women in the general population [22,23,27,28]. Participants were residents of the Australian Capital Territory (ACT) region of Australia, which includes the city of Canberra (population of approximately 314,000 in 2002). All aspects of the study were approved by the ACT Human Research Ethics Committee.

At the first phase of the study, self-report questionnaires were posted to a sample of 10,000 female ACT residents aged 18–42 years, selected at random from the electoral roll and stratified by age in 5-year bands (18–22 years, 23–27 years, 28–32 years, 33–37 years, and 38–42 years) (in Australia, inclusion on the Electoral Roll is a legal requirement for residents aged 18 years or more). The questionnaire included measures of eating disorder symptoms, health-related quality of life, subjective quality of life and socio-demographic information. Body mass index (BMI, kg/m^2^) was calculated from self-reported height and weight. In pilot work, we found a very high correlation (*r* = .97) between BMI calculated in this way and BMI calculated according to actual (measured) height and weight [29].

Completed questionnaires were received, following reminder letters, from 5,255 individuals, which represented a response rate of 57.1% after incorrectly listed addresses (*n* = 684) and individuals away from home at the time of the survey (*n* = 112) were taken into account. This is a conservative estimate of true response because not all individuals with incorrectly listed addresses would have been identified [30]. The sample comprised approximately 10% of the total population of women aged 18 to 42 years in the ACT region and was representative of this population on a range of socio-demographic variables, including marital status, employment status, highest level of education completed, parity, and first language [28].

The ACT is a highly urbanized region and this was reflected in the characteristics of participants. Thus, most participants (85.3%) were born in Australia, had English as their first language (91.8%) and had completed 12 or more years of formal education (90.5%). A majority of participants (55%) were married or living as married, 43.8% had one or more children, 62.8% were employed full- or part-time, 15.6% were full-time students and 17.5% nominated home duties as their main activity. Participants’ mean (*SD*) age was 30.3 (7.2) years. Their mean (*SD*) BMI was 24.5 (5.3) kg/m^2^.

### Study measures

#### Body dissatisfaction

BD was assessed using two items of the measure of eating disorder symptoms, namely, the Eating Disorder Examination Questionnaire (EDE-Q). The EDE-Q [31] is a widely-used, 36-item self-report measure that focuses on the past 28 days. Subscale scores and a global score may be derived from 22 items that assess key attitudinal features, namely, concerns about dietary intake, concerns about eating, concerns about weight and concerns about shape, whereas remaining items assess the occurrence and frequency of eating disorder behaviors, namely, binge eating, self-induced vomiting, misuse of laxatives or diuretics, extreme dietary restriction and excessive exercise [28,29].

Two of the items comprising the EDE-Q weight/shape concerns subscales specifically assess body dissatisfaction, namely, “How dissatisfied have you felt about your weight” and “How dissatisfied have you felt about your shape”? Response options for these items range from 0 to 6 with higher scores indicating greater dissatisfaction and with descriptors indicating that scores of “0”, “2”, “4” and “6” correspond to being “not at all”, “slightly”, “moderately” and “markedly” dissatisfied, respectively. Since scores on these items were highly correlated (*r* = 0.89), a single “dissatisfaction with weight or shape” score was obtained as the simple average of scores on the items concerned.

#### Health-related quality of life

Health-related quality of life was assessed using the Medical Outcomes Study (12-item) Short-Form disability scale (SF-12) [32]. Items of the SF-12 are summarised into two weighted scales (Physical Component Summary scale, PCS; Mental Component Summary scale, MCS), designed to assess physical and mental health impairment. Each scale is scored to have a mean of 50 and standard deviation of 10, with lower scores indicating higher levels of impairment. The SF-12 has very good psychometric properties, including demonstrated validity in the Australian population [3,26,32]. PCS items include, for example, “Does your health now limit you in moderate activities, such as moving a table, vacuuming or playing golf?” and “During the past four weeks, were you limited in the kind or work or other activities undertaken as a result of your physical health?”, whereas MCS items include, for example, “During the past four weeks have you accomplished less than you would like as a result of any emotional problems?” and “During the past four weeks how much of the time have you felt calm and peaceful”? The item response scheme is variable across the items, some items being dichotomous and others having 3, 5 or 6 response options.

#### Subjective quality of life

Subjective quality of life was assessed using the World Health Organization Brief Quality of Life Assessment Scale (WHOQOL-BREF), a 26-item measure yielding scores on each of four domains relating to the individual’s subjective evaluation of his/her physical health, environmental health, psychological functioning and social relationships [33,34]. Items are scored on a five-point, Likert-type scale, with scores of “1” and “5” indicating, respectively, extreme dissatisfaction and extreme satisfaction. Only items comprising the Psychological Functioning (QOL-P; 6 items) and Social Relationships (QOL-S; 3 items) domains were included in the *Health and Well*-*Being Study*[35,36]. Items of the QOL-P include, for example, “To what extent do you feel your life to be meaningful”? and “How satisfied are you with yourself?’, whereas items of the QOL-S include, for example, “How satisfied are you with your personal relationships”? and “How satisfied are you with the support you get from your friends?” One of the QOL-P items, which explicitly addresses satisfaction with bodily appearance, was excluded from the analysis, whereas an additional item of the WHOQOL-BREF, which assesses overall satisfaction with quality of life, and which does not contribute to subscale scores, was included.

### Statistical analysis

Since averaging scores on the items assessing BD dissatisfaction with weight and shape had the effect of introducing non-integer values (i.e., 0.5, 1.5, 2.5, etc.), scores on the BD variable were first recoded so as to preserve the original (7-point) ordinal coding scheme. That is, scores of “0.5” and “1.5” were recoded to “1”, scores of “2.5” and “3.5” were recoded to “3”, and so on. This transformation would have had little impact, since most participants (69.2%) had the same score on the original (“dissatisfaction with weight”, “dissatisfaction with shape”) items.

Both summary-scale and item-level analysis were conducted to examine the associations between BD and impairment in specific aspects of quality of life. Bivariate associations between BD and scores on summary scales measures, namely, the SF-12 PCS and MCS and WHOQOL-BREF QOL-P and QOL-S, were calculated using the Spearman rank order correlation coefficient (Spearman’s rho), whereas analysis of variance was used to compare mean summary scale scores on each measure between subgroups of participants reporting different levels of body dissatisfaction. For analysis involving PCS and MCS summary scores, both the standard scoring method, employing factor scores derived by means of orthogonal factor rotation, and an alternative method, employing factor scores derived by means of oblique factor rotation, were employed [37]. Since the results were unchanged, only findings based on the standard scoring method are reported. For the item-level analysis, a series of ordinal logistic regression analyses was conducted, with each of the SF-12 and WHOQOL-BREF items as outcome variables, in order to examine the likelihood of reporting quality of life impairment for participants with each of the 6 non-zero levels of BD (BD1–BD6) relative to participants with no BD (BD0).

To address the possibility that any observed associations between BD and quality of life impairment were due to an association between BD and eating disorder symptoms, the regression analysis was repeated controlling for the occurrence of eating disorder symptoms. Participants with eating disorder symptoms (“probable eating disorder cases”) were identified using an operational definition employed in previous, population-based research, namely, the “undue influence of weight or shape on self-evaluation” in conjunction with the regular occurrence of any eating disorder (binge eating or extreme weight-control) behavior [23,38]. The “undue influence of weight or shape on self-evaluation”, which is correlated with but distinct from BD [39] and which is included among the diagnostic criteria for both anorexia nervosa and bulimia nervosa [40], was defined as a score of 5 or 6 on either or both of the two EDE-Q items that assess this construct [23,41]. For binge eating, self-induced vomiting and misuse of laxatives or diuretics, “regular” was defined as “at least weekly”. “Regular extreme dietary restriction” was defined as “going without food for a period of 8 or more waking hours to influence weight or shape, on average, three or more times per week”, whereas “regular excessive exercise” was defined as “exercising hard to influence weight or shape, on average, five or more times per week” [28]. This operational definition has been found to identify a highly symptomatic subgroup of women, in terms of eating disorder and comorbid psychopathology, in previous, population-based research [23,38].

Covariates included in all analysis were: age; BMI; marital status; employment status; educational attainment; parity (children, no children); first language (English, not English); country of birth (Australia, not Australia); and (as a proxy for income) possession of private health insurance. Complete case analysis (listwise deletion of missing values) was employed. Levels of missing data were < 2.0% for the variables considered in the current study, with the exception of the BMI variable for which data were missing for 363 participants (6.9%). The maximum effective sample size for the current study analyses was, therefore, 4,892 [16,23]. A significance level of .05 was used for all tests and all analysis was conducted using the Statistical Package for the Social Sciences (SPSS) v.21.0.

## Results

As can be seen in Table 1, BD was common in this sample with most participants (86.9%) reporting some degree of dissatisfaction with their weight or shape and more than one third (36.6%) reporting moderate to marked dissatisfaction.

**Table 1:** **Mean (*SE*) scores on measures of health-related quality of life (SF-12 Physical and Mental Component Summary scales; SF-12 PCS, MCS) and subjective quality of life (WHOQOL-BREF Psychological and Social Functioning subscales; QOL-P, QOL-S) among participants (n=4,892) reporting each of 7 levels of body dissatisfaction (BD0-BD6)**^**i**^

	**Not at all dissatisfied**	**Not at all/ slightly dissatisfied**	**Slightly dissatisfied**	**Slightly/ moderately dissatisfied**	**Moderately dissatisfied**	**Moderately/ markedly dissatisfied**	**Markedly dissatisfied**				
	**(BD0)**	**(BD1)**	**(BD1)**	**(BD1)**	**(BD1)**	**(BD1)**	**(BD1)**				
*n*	674	1264	647	687	594	598	695				
%	13.1	24.5	12.5	13.3	11.5	11.6	13.5				
	**Mean (*SE*)**	**Mean (*SE*)**	**Mean (*SE*)**	**Mean (*SE*)**	**Mean (*SE*)**	**Mean (*SE*)**	**Mean (*SE*)**	***F***	***p***	**Post-Hoc**^**ii**^	**Effect Size**^**iii**^
SF-12 PCS	49.63 (.38)	49.79 (.27)	50.41 (.36)	50.46 (.35)	50.10 (.39)	49.75 (.38)	48.64 (.38)	2.74	< .05	2,3>6	.01
SF-12 MCS	50.90 (.46)	49.04 (.33)	47.44 (.44)	44.79 (.43)	45.47 (.47)	41.25 (.46)	38.59 (.47)	81.47	< .01	0>1,2>3,4>5>6	.11
QOL-P	3.98 (.03)	3.82 (.02)	3.77 (.03)	3.63 (.02)	3.60 (.03)	3.38 (.03)	3.14 (.03)	107.57	< .01	0>1,2>3,4>5>6	.14
QOL-S	4.08 (.03)	3.92 (.02)	3.85 (.03)	3.71 (.03)	3.65 (.04)	3.48 (.03)	3.33 (.03)	53.21	< .01	0>1,2,3>4>5>6; 1>3	.07

^i^Lower scores indicate higher levels of impairment on all measures. Means were adjusted for the following covariates: age, BMI; marital status; employment status; level of education; parity; first language, country of birth and possession of private health insurance.

^ii^Post-hoc tests were adjusted using the Bonferroni correction.

^iii^Partial eta-squared: values of approximately .01, .06, and .14, indicate, small, medium, and large effect sizes, respectively [42].

Also shown in Table 1 are mean scores on each of the four summary QoL measures for participants reporting different levels of BD. As can be seen, higher levels of BD were associated with lower scores on all four summary scale measures, SF-12 PCS, SF-12 MCS, QOL-P and QOL-S, although the effect size for the difference between groups on the SF-12 PCS was small. The rank order correlations between BD and scores on SF-12 PCS, SF-12 MCS, QOL-P, and QOL-S, were, respectively, -.10, -.30, -.36 and -.26. The correlation between BD and BMI was .47.

Results of the ordinal logistic regression analyses are summarised in Table 2. As can be seen, all levels of BD were associated with impairment in at least some aspects of quality of life, after controlling for BMI and other potential covariates. Further, the number of items for which BD was associated with increased likelihood of quality of life impairment increased proportional to the level of BD reported.

**Table 2:** **Results of ordinal logistic regression analyses showing odds ratios (*OR*s) and confidence intervals (*CI*s) for poorer quality of life on items of the WHOQOL-BREF and SF-12, according to participants’ (n=4,892) level of body dissatisfaction (BD) **^**i-iii**^

	**BD1**	**BD2**	**BD3**	**BD4**	**BD5**	**BD6**
	***OR* (95% *CI)***	***OR* (95% *CI)***	***OR *(95% *CI)***	***OR *(95% *CI)***	***OR *(95% *CI)***	***OR *(95% *CI)***
**WHOQOL**-**BREF items**						
*Psychological Functioning*^*iv*^						
Positive Feelings	1.22 (0.98, 1.52)	1.49 (1.18, 1.87)**	1.86 (1.44, 2.41)***	2.02 (1.59, 2.58)***	3.23 (2.47, 4.21)***	4.04 (3.12, 5.22)***
Spirituality	1.45 (1.18, 1.78)***	1.70 (1.37, 2.11)***	2.27 (1.78, 2.89)***	2.54 (2.02, 3.20)***	3.39 (2.63, 4.36)***	4.98 (3.90, 6.36)***
Thinking	1.29 (1.04, 1.59)*	1.37 (1.10, 1.70)**	1.80 (1.40, 2.32)***	2.00 (1.58, 2.53)***	2.93 (2.26, 3.79)***	4.21 (3.28, 5.41)***
Self-esteem	1.84 (1.47, 2.30)***	2.60 (2.07, 3.27)***	3.89 (3.01, 5.04)***	5.00 (3.91, 6.38)***	9.35 (7.15, 12.23)***	24.58 (18.83, 32.07)***
Negative feelings	1.52 (1.22, 1.88)***	1.72 (1.37, 2.15)***	2.41 (1.87, 3.10)***	2.83 (2.23, 3.59)***	4.45 (3.43, 5.77)***	8.47 (6.56, 10.94)***
*Social Relationships*^*v*^						
Personal relationships	1.53 (1.24, 1.89)***	1.64 (1.32, 2.04)***	2.18 (1.70, 2.78)***	2.81 (2.23, 3.54)***	3.79 (2.93, 4.89)***	4.33 (3.38, 5.55)***
Sexual activity	1.34 (1.10, 1.65)**	1.68 (1.36, 2.07)***	1.91 (1.51, 2.42)***	2.39 (1.91, 2.99)***	3.22 (2.52, 4.11)***	3.78 (2.98, 4.81)***
Social support	1.26 (1.02, 1.54)*	1.63 (1.32, 2.02)***	1.94 (1.53, 2.47)***	2.06 (1.64, 2.58)***	2.63 (2.05, 3.38)***	3.81 (2.99, 4.85)***
*Overall quality of life*^*vi*^	1.26 (1.02, 1.57)*	1.38 (1.10, 1.72)**	1.69 (1.31, 2.18)***	2.06 (1.62, 2.62)***	2.55 (1.96, 3.31)***	3.60 (2.78, 4.65)***
**SF**-**12 items**						
*Physical Health*^*vii*^						
General health	1.25 (1.02, 1.53)*	1.37 (1.11, 1.69)**	1.52 (1.20, 1.93)**	1.90 (1.51, 2.38)***	2.17 (1.70, 2.77)***	3.75 (2.95, 4.77)***
Accomplish less	1.00 (0.78, 1.28)	1.01 (0.79, 1.31)	1.34 (1.01, 1.77)*	1.40 (1.07, 1.82)*	1.43 (1.07, 1.91)*	2.44 (1.86, 3.21)***
Limited in kind	1.00 (0.77, 1.31)	1.08 (0.82, 1.42)	1.00 (0.73, 1.36)	1.12 (0.84, 1.50)	1.43 (1.06, 1.95)*	1.73 (1.30, 2.32)***
Moderate activities	0.71 (0.53, 0.95)*	0.67 (0.49, 0.91)*	0.62 (0.44, 0.89)**	0.64 (0.46, 0.89)**	0.87 (0.62, 1.22)	1.03 (0.75, 1.41)
Climb several flights	0.87 (0.65, 1.16)	0.82 (0.61, 1.11)	0.91 (0.66, 1.27)	0.95 (0.70, 1.29)	1.30 (0.94, 1.78)	1.58 (1.17, 2.14)**
Pain-interfere	1.23 (1.00, 1.52)	1.20 (0.97, 1.49)	1.36 (1.07, 1.74)*	1.59 (1.27, 2.01)***	1.51 (1.17, 1.95)**	2.12 (1.66, 2.70)***
*Mental Health*^*viii*^						
Accomplish less	1.23 (0.95, 1.60)	1.77 (1.36, 2.31)***	2.64 (1.98, 3.54)***	2.33 (1.77, 3.08)***	4.10 (3.05, 5.52)***	6.25 (4.67, 8.36)***
Not careful	1.21 (0.91, 1.60)	1.62 (1.21, 2.16)**	2.57 (1.88, 3.50)***	2.29 (1.70, 3.08)***	3.53 (2.58, 4.82)***	5.09 (3.75, 6.89)***
Peaceful	1.53 (1.24, 1.87)***	1.95 (1.58, 2.42)***	2.62 (2.06, 3.32)***	2.94 (2.35, 3.69)***	4.34 (3.40, 5.56)***	6.71 (5.27, 8.55)***
Energy	1.25 (1.02, 1.53)*	1.50 (1.22, 1.85)***	2.09 (1.65, 2.64)***	2.36 (1.89, 2.95)***	3.98 (3.11, 5.08)***	5.95 (4.68, 7.56)***
Blue/sad	1.57 (1.27, 1.94)***	1.86 (1.49, 2.32)***	2.51 (1.96, 3.22)***	2.78 (2.20, 3.50)***	4.55 (3.53, 5.87)***	7.49 (5.85, 9.61)***
Social-time	1.12 (0.90, 1.39)	1.33 (1.06, 1.66)*	1.96 (1.53, 2.52)***	1.71 (1.35, 2.16)***	2.55 (1.98, 3.28)***	4.163.25, 5.34)***

^i^For all items, odds ratios greater than 1.0 indicate increased likelihood of greater quality of life impairment for the item concerned for participants reporting non-zero levels of body dissatisfaction (BD1 – BD6), relative to participants who reported no body dissatisfaction (BD0). BD1 = none/slight body dissatisfaction; BD2 = slight body dissatisfaction; BD3 = slight/moderate body dissatisfaction; BD4 = moderate body dissatisfaction; BD5 = moderate/marked body dissatisfaction; BD6 = marked body dissatisfaction.

^ii^Odds ratios were adjusted for the following covariates: age, BMI; marital status; employment status; level of education; parity; first language, country of birth and possession of private health insurance.

^iii^*** *p* < 0.001, ** *p* < 0.01, * *p* < 0.05.

^iv^WHOQOL-BREF *Psychological Functioning Subscale* (*QOL*-*P*) *items*.

Positive Feelings: How much do you enjoy life? (Not at all; A little; A moderate amount; Very much; An extreme amount).

Spirituality: To what extent do you feel your life to be meaningful? (Not at all; A little; A moderate amount; Very much; An extreme amount).

Thinking: How well are you able to concentrate? (Not at all; A little; A moderate amount; Very much; Extremely).

Self-esteem: How satisfied are you with yourself? (Very dissatisfied; dissatisfied; Neither satisfied nor dissatisfied; Satisfied; Very satisfied).

Negative feelings: How often do you have negative feelings such as blue mood, despair, anxiety, depression? (Never; Seldom; Quite often; Very often; Always).

^v^WHOQOL-BREF *Social Relationships Subscale* (*QOL*-*S*) *items*.

Personal relationships: How satisfied are you with your personal relationships? (Very dissatisfied; dissatisfied; Neither satisfied nor dissatisfied; Satisfied; Very satisfied).

Sexual activity: How satisfied are you with your sex life? (Very dissatisfied; dissatisfied; Neither satisfied nor dissatisfied; Satisfied; Very satisfied).

Social support: How satisfied are you with the support you get from your friends? (Very dissatisfied; dissatisfied; Neither satisfied nor dissatisfied; Satisfied; Very satisfied).

^vi^WHOQOL-BREF *Overall quality of life* item: How would you rate your quality of life? (Very poor; Poor; Neither poor nor good; Good; Very good).

^vii^SF-12 *Physical Component Summary Scale* (*PCS*) *items*.

General health: In general, would you say your health is …? (Excellent; Very good; Good; Fair; Poor).

Accomplish less: During the past four weeks, have you accomplished less than you would like as a result of your physical health? (Yes, No).

Limited in kind: During the past four weeks, were you limited in the kind or work or other regular activities you do as a result of your physical health? (Yes, No).

Moderate activities: Does your health now limit you in moderate activities, such as moving a table, pushing a vacuum cleaner, bowling or playing golf? (Yes, limited a lot; Yes, limited a little; No, not limited at all).

Climb several flights: Does your health now limit you in climbing several flights of stairs? (Yes, limited a lot; Yes, limited a little; No, not limited at all).

Pain-interfere: During the past four weeks, how much did pain interfere with your normal work (including both work outside the home and housework)? (Not at all; A little bit; Moderately; Quite a bit; Extremely).

^viii^SF-12 *Mental Component Summary Scale* (*MCS*) *items*.

Accomplish less: During the past four weeks, have you accomplished less than you would like to as a result of any emotional problems, such as feeling depressed or anxious)? (Yes, No).

Not careful: During the past four weeks, did you not do work or other regular activities as carefully as usual as a result of any emotional problems (such as feeling depressed or anxious? (Yes, No).

Peaceful: How much of the time during the past four weeks have you felt calm and peaceful? (All of the time; Most of the time; A good bit of the time; Some of the time; A little of the time; None of the time).

Energy: How much of the time during the past four weeks did you have a lot of energy? (All of the time; Most of the time; A good bit of the time; Some of the time; A little of the time; None of the time).

Blue/sad: How much of the time during the past four weeks have you felt downhearted and blue? (All of the time; Most of the time; A good bit of the time; Some of the time; A little of the time; None of the time).

Social-time: During the past four weeks, how much of the time has your physical health or emotional problems interfered with your social activities (like visiting with friends, relatives, etc.)? (All of the time; Most of the time; Some of the time: A little of the time; None of the time).

As is also apparent in Table 2, increased likelihood of quality of life impairment associated with BD was more likely to be observed for items of the SF-12 tapping mental health than those tapping physical health, although greater BD was strongly associated with increased likelihood of impairment for certain aspects of physical health. In particular, participants who reported marked BD were 3.75 times more likely to report poorer perceived general health than those who reported no BD, after controlling for age, BMI and socio-demographic characteristics.

As would be expected, individuals with eating disorder symptoms (n=482, 9.2%) were over-represented among participants with moderate (BD = 5: 19.2%) and marked (BD = 6: 43.3%) BD, whereas the prevalence of eating disorder symptoms was low in the remainder of the study population, ranging from 0.4% among participants with no BD to 3.8% among participants with moderate BD (*χ*^*2*^ = 1300.0, p < .01). As would also be expected, effect sizes for some associations were reduced when the regression analysis was repeated controlling for the occurrence of eating disorder symptoms. For example, the odds ratio for the SF-12 PCS *General Health* item for participants who reported marked BD changed from 3.75 to 3.50, whereas the odds ratio for the SF-12 MCS *Blue*/*Sad* item for participants who reported marked BD changed from 7.49 to 5.79. However, the pattern of findings was unchanged and all previously significant effects remained significant.

## Discussion

### Summary of main findings

We examined impairment in health-related and subjective quality of life associated with BD in a large, general population sample of women. Most participants (86.9%) reported some level of dissatisfaction with their weight or shape and more than one third (39.4%) reported moderate to marked dissatisfaction. Higher levels of BD were associated with poorer quality of life for all items of both quality of life measures, the degree of impairment being proportional to the degree of dissatisfaction. Associations were strongest for items tapping mental health and psychosocial functioning, although greater BD was associated with substantially increased risk of impairment in certain aspects of physical health even when controlling for body weight. Post-hoc analysis suggested that the observed associations between BD and quality of life impairment were not due to an association between BD and eating disorder symptoms.

### Study implications

To our knowledge, this is the first study to consider impairment in quality of life associated with BD in a large, general population sample of women. The most notable finding was that BD was associated with marked impairment in various aspects of quality of life in a substantial proportion of participants. This finding is notable because interest in BD has, thus far, been largely confined to its role as a risk factor for “more adverse” outcomes, such as low self-esteem, depressive mood and eating disorder symptoms [13,17,18]. When both the prevalence of BD and the degree of associated impairment are considered, it is apparent that there is a very substantial public health burden of BD at the population level. Hence, the present findings suggest that greater attention may need to be given to BD as a public health problem in its own right. Some, tentative steps in this direction are now being taken, for example, in the form of government-sanctioned, though voluntary, regulations relating to the depiction of body image in the popular media [17], but there is little in the way of a co-ordinated, public health approach. We hope that the present findings will serve as an incentive for action in this regard. An additional implication of the present findings is that the fact that dissatisfaction with weight or shape is “normative” in industrialized nations should not be taken to infer that it is benign.

As expected, impairment in quality of life associated with BD was more likely to be observed for items tapping mental health and psycho-social functioning than for those tapping physical health status. Nevertheless, moderate to marked BD was found to be associated with substantially increased risk of impairment in certain aspects of physical health even after controlling for age, body weight and socio-demographic characteristics. The explanation for these latter associations is unclear. Muennig and colleagues [21], who similarly found, in a general population sample of women and men, that BD was independently associated with both physical and mental health impairment, suggested that stress associated with negative body image may mediate the association between BD and physical health impairment. Alternatively, or in addition, personality characteristics associated with BD, such as low self-esteem, depressive mood and perfectionism, may be conducive to an unduly negative appraisal of physical health [43]. Along similar lines, it would not be surprising, given the pervasiveness of current public health messages concerning the adverse health consequences of obesity, to find that at least some women who are dissatisfied with their bodies believe themselves to be overweight and/or “unhealthy” when in fact they may be neither [44-46]. Finally, BD may be associated with lower levels of physical activity, hence poorer physical health, due to avoidance of bodily exposure [47,48].

Because the links between BD and eating disorder symptoms are particularly strong [18], and because there is good evidence that eating disorder symptoms are associated with marked impairment in quality of life in women [22,23], post-hoc analysis was conducted to address the possibility that the impairment in quality of life associated with BD observed in the present study might have been due, at least in part, to an association between BD and eating disorder symptoms. However, there was little evidence to support this hypothesis. Effect sizes for certain associations were marginally reduced when the original analysis was repeated controlling for the occurrence of eating disorder symptoms (among participants with high levels of BD). Otherwise, the findings were unchanged.

The latter finding is notable because eating disorder prevention programs have generally been more successful in reducing the occurrence of BD and related constructs than eating disorder symptoms per se [49]. The present findings suggest that health promotion programs that are successful in reducing the occurrence of BD are likely to have substantial benefits in terms of individual and community well-being irrespective of whether they are successful in reducing the occurrence of eating disorder symptoms. In our view, both BD and eating disorder symptoms warrant greater attention as public health problems, particularly in the context of obesity prevention [12,16,23]. However, BD may be the more rational target for health promotion efforts given that it is: (i) a potent risk factor for various adverse health outcomes; (ii) common; and (iii) associated with considerable distress and disability in its own right.

### Study limitations and other methodological considerations

At least three limitations of the present study need to be considered when interpreting the findings. First, the assessment of BD was confined to two items assessing participants’ subjective dissatisfaction with their weight or shape. The advantage of this assessment was that it encompassed a brief, relatively unambiguous measure suitable for use in an epidemiological study. However, it would be helpful to replicate the present findings using a more sophisticated, multidimensional measure of BD [12,50].

Second, the generalizability of the present findings is constrained by the choice of study population, namely, young adult women from an urbanized and comparatively affluent region of Australia. Hence, replication of the present findings in women of different ages and in women and men from more diverse backgrounds would be a useful contribution to future research [12,15,51]. The demonstration of similar associations in other study populations would further strengthen the case for recognition of BD as a public health problem. Replication of the present study method in a population-based sample of men would be of particular interest. Although anecdotal evidence has been taken to infer that the prevalence of BD and/or its impact on mental health may be increasing in men, until recently there has been little in the way of empirical evidence to support either contention [12,52].

Third, the method employed to determine that the observed associations between BD and quality of life impairment were not accounted for by an association between BD and eating disorder symptoms, namely, controlling for participants with a high levels of eating disorder symptoms (“probable eating disorder cases”), cannot be considered definitive. Since eating disorder symptoms, like BD, occur on a continuum, it is possible that the occurrence of lower levels of symptomatology among remaining participants influenced the observed associations between BD and quality of life. Although it would have been possible to employ the EDE-Q global score as continuous covariate, this course was not taken because the EDE-Q global score is primarily a measure of concerns about weight, shape and eating and as such is highly correlated with the items that assess BD (r = 0.85 in the present study population) [28,29]. By definition, eating-disordered behavior entails, in addition to attitudinal features such as the “undue influence of weight or shape on self-evaluation”, the regular occurrence of one or more eating disorder behaviors [53].

Finally, this was a cross-sectional study. Conceivably, poor quality of life could be conducive to BD or associations might exist in both directions [54]. Although the direction of the observed associations cannot be determined on the basis of the present study, it may be noted that findings from prospective epidemiological studies strongly support the role of BD in predicting adverse health outcomes, whereas evidence for the role of low self-esteem, depressive mood and other such outcomes in predicting BD is less compelling [13,54]. Notable strengths of the current study were the recruitment of a large, general population sample of women, the inclusion of two different, widely-used measures of quality of life and the assessment of eating disorder symptoms in addition to BD.

## Conclusions

In women, BD is associated with marked impairment in aspects of quality of life relating to mental health and psycho-social functioning and at least some aspects of physical health, independent of its association with body weight and eating disorder symptoms. Given this, and given its high prevalence, greater attention may need to be given to BD as a public health problem. The fact that BD is “normative” should not be taken to infer that it is benign.

## Ethics approval

The research was conducted with the approval of the ACT Human Research Ethics Committee.

## Competing interests

The authors declare that they have no competing interests.

## Authors’ contributions

JM was responsible for the design and conduct of the research as well as data processing, initial data analysis and manuscript preparation. DM conducted supplementary data analysis and assisted with interpretation of this analysis. BR, PH and CO contributed to the design and conduct of the research and to critical revision of an earlier version of the manuscript. BR contributed to data analysis and interpretation. All authors read and approved the final manuscript.

## Pre-publication history

The pre-publication history for this paper can be accessed here:

http://www.biomedcentral.com/1471-2458/13/920/prepub
